# Sensory adaptation in the barrel cortex during active sensation in the behaving mouse

**DOI:** 10.1038/s41598-024-70524-1

**Published:** 2024-09-16

**Authors:** Andrea Colins Rodriguez, Michaela S. E. Loft, Ingo Schiessl, Miguel Maravall, Rasmus S. Petersen

**Affiliations:** 1 Division of Neuroscience, School of Biological Sciences, Faculty of Biology, Medicine and Health, University of Manchester, Manchester, M13 9PT UK; 2 Geoffrey Jefferson Brain Research Centre, Manchester Academic Health Science Centre, Northern Care Alliance NHS Foundation Trust, University of Manchester, Manchester, M6 8HD UK; 3 Sussex Neuroscience, School of Life Sciences, University of Sussex, Brighton, BN1 9RH UK

**Keywords:** Sensorimotor processing, Sensory processing, Barrel cortex, Whisker system

## Abstract

Sensory Adaptation (SA) is a prominent aspect of how neurons respond to sensory signals, ubiquitous across species and modalities. However, SA depends on the activation state of the brain and the extent to which SA is expressed in awake, behaving animals during active sensation remains unclear. Here, we addressed this question by training head-fixed mice to detect an object using their whiskers and recording neuronal activity from barrel cortex whilst simultaneously imaging the whiskers in 3D. We found that neuronal responses decreased during the course of whisker-object touch sequences and that this was due to two factors. First, a motor effect, whereby, during a sequence of touches, later touches were mechanically weaker than early ones. Second, a sensory encoding effect, whereby neuronal tuning to touch became progressively less sensitive during the course of a touch sequence. The sensory encoding effect was whisker-specific. These results show that SA does occur during active whisker sensing and suggest that SA is fundamental to sensation during natural behaviour.

## Introduction

Sensory Adaptation (SA), first demonstrated in single neurons by Adrian & Zotterman in 1926^1^, is exemplified by the phenomenon that the response of neurons to a sensory stimulus of fixed strength (e.g., whisker deflection or light flash) progressively decreases during the course of a sequence of such stimuli. In general, SA refers to phenomena where neuronal responses are dynamic – depending not only on the immediate stimulus but also on the temporal context in which they are embedded^2–14^.

SA has been thoroughly studied in a ‘passive stimulation’ paradigm, where stimuli are applied to an immobilised animal – the animal is the passive recipient of stimulation^2,4,5,10,14–20^. This paradigm is experimentally convenient for studying SA, since the experimenter has a high degree of control over the sensory stimuli. However, the experimental conditions differ from those of behaving animals in important respects.

Behaving animals acquire sensory information by making exploratory movements of their sense organs (active sensing)^21,22^. For example, primates explore scenes by eye movements and rats/mice by whisker movements. The value of active sensing is that it allows animals to make actions that sample sensory information most useful to the animal’s current goals and thus to attain those goals more efficiently than would be possible via passive sensing^23^. Human eye movements can approach the efficiency of an ideal Bayesian planner^24^ and rats/mice use whisker movements to turn challenging tactile discrimination tasks into more straightforward detection tasks^25,26^. However, it is technically challenging to study SA during active sensing. In contrast to the passive stimulation paradigm, where SA can be straightforwardly assayed by comparing the response of neurons to a sequence of stimuli of fixed strength, during active sensing, animals continually move their sense organs and thereby change the sensory input. This presents a significant difficulty for the investigation of SA: a change in neuronal response during the course of a sequence of whisker touches might not be due to SA but rather to a change in touch strength. Whether SA is expressed in the actively sensing animal engaged in a goal-driven task is an open question.

Here we address this question by studying the mouse whisker system and taking advantage of recent advances in methods for quantifying whisker-dependent behaviour^25,27,28^. The whisker system exhibits active sensing in a tractable form: rats/mice explore objects by probing them with back-and-forth movements of their whiskers (“whisking”). There is consistent evidence for SA in the whisker-related zone of primary somatosensory cortex of the anaesthetised rat/mouse^4,5,15–19,29,30^ and this SA is specific to individual whiskers^17,31–36^. Using the passive stimulation paradigm, there is also evidence for SA in awake mice^10,20,32–37^. However, SA has not been studied in awake rats/mice during active touch.

To test whether SA occurs in the behaving, actively sensing mouse, we trained mice to perform a whisker-guided object detection task, and then recorded neural activity from barrel cortex whilst, simultaneously, we imaged their whiskers in 3D. These whisker measurements allowed us to disentangle motor effects on neural activity from sensory ones. We found that cortical responses to whisker-object touch do depend on prior touches. This effect originates not only from a direct effect on the responsiveness of sensory neurons (SA) but also from an indirect effect whereby touch triggers a change in motor action which in turn affects the strength of future touches.

## Methods

All experiments were done in accordance with the UK Animals (Scientific Procedures) Act (1986). All experimental protocols described in here were approved by both United Kingdom Home Office national authorities and institutional ethical review, and are in accordance with the ARRIVE guidelines.

### Behavioural apparatus

Whiskers were imaged similarly to^28^ using two high-speed cameras (LTR2, Mikrotron, Unterschleissheim, Germany; 480 × 480 pixels, pixel width 0.047 mm; 1000 frames/s, 0.2 ms exposure time) with telecentric lenses (55–349, Edmunds Optics, Barrington, NJ). In relation to standard anatomical planes for the skull, one camera imaged the horizontal plane; the second camera imaged a plane 25^◦^ off the sagittal plane and 10^◦^ off the horizontal plane, chosen to minimise occlusion between the cheek of the mouse and the whiskers. Mice were head-fixed and positioned inside a perspex tube (inner diameter 32 mm). The whiskers were free to move at all times. Whiskers were illuminated from below using an infrared LED array (940 nm, LED 940–66-60, Roithner, Vienna, Austria) via a diffused condenser lens.

### Surgical procedure and mice training

Mice (C57; males; N = 11; 6 weeks at the time of implant) were implanted with a titanium head-bar under isoflurane anaesthesia (for details, see^38^). For all recovery procedures in this study, Buprenorphine (0.1 mg/kg) was injected for postoperative analgesia. After surgery, mice were left to recover for at least 5 days before starting water restriction (1.5 ml water/day). Training began 7–10 days after the start of water restriction. Mice were trained for water reward until they achieved a success rate of 70% on the task defined below. Subsequently, a craniotomy was performed as detailed in the following section and sealed with silicone. Mice were left to recover for 2–3 days until performance had recovered before the first recording session.

### Electrophysiological recordings

Mice were trimmed to 3 whiskers in C row (C1, C2 and C3) and intrinsic optical imaging was performed, whilst deflecting whisker C1 or C2 using a piezoelectric actuator to locate C1 or C2 whisker column, under isoflurane anaesthesia^39^. A craniotomy was then performed at this location and filled with silicone elastomer. At the start of each recording session, the elastomer was removed and a 32-channel linear silicon probe (2 shanks; each shank had a 2 × 8 array of 12 µm recording sites, spanning ~ 200 µm along the long axis of the shank; 250 µm inter-shank spacing, Cambridge NeuroTech, Cambridge, United Kingdom) was inserted into the brain at a rate of approximately 5 μm/s under brief isoflurane anaesthesia so that the depth of the recording sites was 600–800 µm below the pial surface. At the end of a recording session (sampling rate 24.4 kHz, filtering 300–3000 Hz), the microelectrode array was withdrawn, the craniotomy sealed with silicone elastomer, and the mouse returned to its home cage.

### Behavioural task

Mice were trained on the pole detection task detailed in^28^. Briefly, mice were trained and imaged in a dark, sound-proofed enclosure. A head-fixed mouse was placed inside a perspex tube, from which its head emerged at one end. The stimulus object was a 0.2 mm diameter, vertical carbon fibre pole which could be translated in the horizontal plane by stepper motors. To allow vertical movement of the pole into and out of range of the whiskers, the apparatus was mounted on a pneumatic linear slide, powered by compressed air, controlled from MATLAB via a real-time processor. In this way, the pole moved rapidly (~ 0.15 s) into and out of range of the whiskers. Mouse responses were monitored by a lick port located anterior to the mouth. Licks were detected as described in^40^. Each lick port consisted of a metal tube connected to a water reservoir via a computer-controlled solenoid valve. Head-fixed mice were trained to detect the presence of the pole using their whiskers, using behavioural procedures similar to^40^. On each trial, the pole was presented either within reach of the whiskers (‘Go trial’) or out of reach (‘NoGo trial’). At the start of each trial, the computer triggered the pole to move up (travel time ~ 100 ms). The pole stayed up for 1 s, before moving down. On Go trials, the correct response was for the mouse to lick the lick port. Correct responses (hits) were rewarded by a drop of water (~ 10 μl). Incorrect responses on Go trials (not licking, misses) were punished by timeout (3–5 s). On NoGo trials, the correct response was to refrain from licking (correct rejection) and incorrect responses (licking, false alarms) were punished by timeout and tone (frequency 12 kHz).

### Whisker tracking

After calibration of the cameras, whiskers were tracked in 3D from the video^28^. Calibration was performed at the end of each recording session.

The shape of each target whisker was described as a quadratic, 3D Bezier curve ${\varvec{b}}\left( s \right) = \left( {x\left( s \right),y\left( s \right),z\left( s \right)} \right)$, defined by 3 control points, where, $x$, $y$, $z$ are space coordinates and $0 \le s \le 1$ parameterises location along the curve: ${\varvec{b}}\left( {s = 0} \right)$ corresponds to the control point closest to the whisker base and ${\varvec{b}}\left( {s = 1} \right)$ to that furthest from the base.

The intrinsic shape of a quadratic curve is fully described by a curvature function $\kappa_{3D} \left( s \right)$ ^41^:

$$\kappa_{3D} \left( s \right) = \frac{{\left| {\frac{{d{\varvec{b}}\left( s \right)}}{ds} \times \frac{{d^{2} {\varvec{b}}\left( s \right)}}{{ds^{2} }}} \right|}}{{\left| {\frac{{d{\varvec{b}}\left( s \right)}}{ds}} \right|^{3} }}$$

Here $\left| a \right|$ denotes the magnitude (2-norm) of vector $a$. $\kappa_{3D} \left( s \right)$ has units of 1/distance and is the reciprocal of the radius of the circle that best fits the curve at point $s$. For a quadratic curve, in the quasi-static framework, we can apply the standard relationship between bending moment during contacts ($M_{z}$) and a change in whisker curvature^42,43^. Thence it follows that $M_{z}$ is proportional to:

$$\Delta \kappa_{3D} \left( s \right) = \kappa_{3D} \left( s \right) - \kappa_{3D,0} \left( s \right)$$

where $\kappa_{3D} \left( s \right)$ is the curvature of the whisker during contact and $\kappa_{3D,0} \left( s \right)$ is the curvature when the whisker is free from contact and in its resting state. All results presented here were evaluated at $s = 0$ and 5 ms after touch onset.

Bezier curves were visually checked and occasional errors were manually corrected. Touches were manually detected at millisecond resolution with the aid of a customised software as in^28^. This step was the main bottleneck in the analysis. We focused on sessions in which a mouse performed at least 40 Go trials and the simultaneous electrophysiological recordings yielded at least 2 touch-responsive single units. This selection resulted in 6 sessions from 3 mice, containing a total of 443 trials and 1,580,064 frames.

### Electrophysiological data analysis

#### Spike sorting

Single units were isolated from the silicon probe recordings using JRCLUST^44^. Recordings from each electrode shank were processed independently. A putative single unit was selected for further analysis if (1) its activity could be tracked during the whole recording, (2) its inter-spike interval histogram exhibited a clear absolute refractory period and (3) its waveform was biphasic and consistent across channels^38^. After the spike sorting process, we selected all units that showed an increase in their mean firing rate shortly after touch onset for further analysis.

#### ROC analysis

ROC analysis was carried out as in^45^. Responses were measured as spike counts in 30 ms non-overlapping time windows. The hit rate (correct detection of touch) was calculated using response windows starting at touch onset. For first touch, the false positive rate was calculated using ‘noise’ responses sampled between trial onset and first touch. For later touches, ‘noise’ responses were sampled from inter-touch intervals.

#### Tuning curves

To measure tuning curves for ${\Delta }\kappa_{3D}$, we first identified touch onset times and categorised them as belonging to first touch on a trial, second touch, etc. We calculated the mean of ${\Delta }\kappa_{3D}$ in the 5 ms interval following each touch onset. The 5% of touches with greatest absolute value were discarded as outliers. The remaining samples were discretised into 4 equipopulated bins and, for each unit, the mean spike count (calculated in the 30 ms bin starting at touch onset) for each bin was calculated separately for first touch events, second touch events, etc. For each unit, the tuning curve was estimated as the linear correlation between spike count and ${\Delta }\kappa_{3D}$.

#### Testing for sensory adaptation

When a mouse touches an object by active touch, it controls the force of whisker-object contact and the strength of the touches vary (Results, Fig. 2). A change in neuronal response to touch during the course of a sequence of touches might therefore reflect SA and/or a change in the strength of touch. To tease these effects apart, we first measured the strength of each touch in the trial (first, second etc.) as $|{\Delta }\kappa_{3D} |$ and measured the tuning curves separately for each touch. The ordinal number of the touch in the trial was defined irrespective of whisker identity. These tuning curves enabled us to estimate (predict) the response of a unit in the state of second touch, for example, to touches of varying $|{\Delta }\kappa_{3D} |$. To test for SA, we compared the response of units to first touch with that predicted by applying touch strengths measured for first touch through the tuning curves estimated for later touches. This procedure was done using a leave-one-out cross-validation process.

To quantify the contribution of SA to the response attenuation of Fig. 2, we used the following approach. For robustness, this analysis was carried out using the summed activity of each simultaneously recorded population (‘population response’). For each population, we first calculated the median response to first touch ($FR_{1}$) and the median response to later touch ($FR_{later}$). The difference $FR_{1}$-$FR_{later}$ is the response attenuation of Fig. 2. Next, we used the tuning curve approach described above to ask how much of this attenuation could be attributed to SA alone. To this end, we calculated the tuning curve of the population response to $|{\Delta }\kappa_{3D} |$ for later touches. We then used this tuning curve to estimate the median population response to first touch (*FR*_1_^′^). In the complete absence of SA, the tuning curve for later touch matches that for first touch, so that *FR*_1_^′^ is equal to $FR_{1}$ and $\alpha$ = 0 in the following equation:

$$\alpha = \frac{{FR_{1} - FR_{1}^{{\prime }} }}{{FR_{1} - FR_{later} }}$$

At the other extreme, if the response attenuation effect is entirely due to SA, $\alpha$ = 1. The index $\alpha$ thus quantifies the degree to which response attenuation in a given population can be explained by SA.

### Stimulus-specific adaptation

For this analysis, trials that met all the following criteria were selected: at least the first two touches in a trial (‘adapting’) were produced by the same whisker (C1 or C2); at least one subsequent touch (‘test’) was produced by the other whisker; the time of the test touch did not overlap with that of any other whisker. For comparison between test touch and later ($N_{touch} \ge 3$) touch responses, we determined tuning curves for later touches, one for each whisker. These tuning curves were obtained as detailed above, using data for all later touches, except (as a cross-validation procedure) the test stimuli. To quantify changes in the responses to the adapting and test stimuli, we used the index defined in Results (Stimulus-Specific Adaptation, Eq. 1).

## Results

### Variability of whisking behaviour

**Fig. 1:**
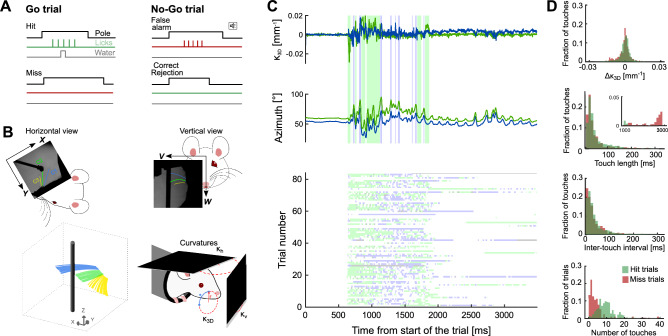
Variability of whisker-pole touches during the pole detection task. (**A**) Behavioural task schematic. Mice were trained to report the pole’s presence within whisker reach (Go trials) by licking a port. On NoGo trials, the pole was moved to a position out of whisker reach and mice should withhold licks. False alarms were penalised with a tone, while Misses were penalised with a time-out period. (**B**) Whisker imaging. Whiskers (C1, C2 and C3) were imaged with two cameras during the behavioural task (top panels). Whiskers were tracked in 3D and 3D curvature ($\kappa_{3D}$) was then calculated (bottom panels). (**C**) Top/Middle: Whisker-pole touches and $\kappa_{3D}$/azimuthal whisker angle from an example Go trial. Bottom: Touches for an example session (in this session only C1 (blue) and C2 (green) whiskers touched the pole). (**D**) Behavioural variability of touches (hit trials green; miss trials red): Strength of touch (${\Delta }\kappa_{3D}$), touch-length (inset shows fraction of touches with contact periods > 1000 ms), inter-touch interval and number of touches per trial. Data from all whiskers and behavioural sessions were included.

Our first aim was to establish a behavioural paradigm to investigate whether Sensory Adaptation (SA) occurs during active touch. Head-fixed mice can discriminate the location of a pole using their whiskers and use active touch to do so^25,26^. As a mouse repeatedly moves its whiskers back and forth against the pole, this results in touch sequences with inter-touch intervals of up to about 100 ms^25,26^. It is well-established that sequences of touches with such intervals, delivered passively to anaesthetised mice, produce strong SA in barrel cortex^15,30^. We therefore reasoned that, if SA is expressed in the awake, actively sensing animal, then the process should be engaged by such behaviour. To test this, mice (N = 11) were trained to solve a Go-NoGo pole detection task (Fig. 1A) with a single row of whiskers (C1, C2 and C3). On ‘Go’ trials, the pole was moved within reach of the whiskers: if the mouse licked the lickport (hit), the mouse was rewarded with a water droplet; if not (miss), it was penalised with a time-out. On ‘NoGo’ trials, the pole was placed out of reach of the whiskers: if the mouse withheld licking (correct rejection), the task proceeded immediately to the next trial; if not (false alarm) the mouse was penalised with a time-out and sound. Mice learned to perform the task accurately (81 ± 17%, mean ± SD over mice), performing 135 ± 22 trials per session. Use of this task allowed us to record a substantial number of trials to achieve good statistical power and to test if SA might be decision-dependent.

In order to measure whisking behaviour in 3D, we imaged the whiskers at 1000 frames/s with two video cameras (Fig. 1B, top). Using a 3D whisker tracking algorithm^28^, both 3D whisker shape and 3D whisker position were extracted from the imaging data (total of 1.8 M frames). From whisker shape, the 3D curvature ($\kappa_{3D}$) at the base of each whisker was estimated for each frame (Fig. 1B, bottom). We estimated the strength of whisker-pole touch in each frame as the change in curvature at the whisker base relative to its intrinsic curvature (${\Delta }\kappa_{3D}$).

As desired, each trial consisted of a sequence of one or more ‘touches’ (a sequence of frames in which the whisker is in continuous contact with the pole) (Fig. 1C). In contrast to the regular stimulus sequences usually used in sensory physiology, we found that the temporal pattern of touches varied substantially across trials. This variability was manifest in number of touches per trial, interval between touch onsets, duration of touch, strength of touch (${\Delta }\kappa_{3D}$) and direction of touch (sign of curvature change) (Fig. 1D). Touches on miss trials were significantly weaker (*t*-test, p < 0.005), longer (*t*-test, p < 10^–10^), sparser (*t*-test, p < 10^–10^) and there were considerably fewer touches on miss trials than on hit trials (10.5 ± 5.7 per trial on hit trials, 5 ± 7.7 on miss trials; *t*-test, p < 10^–10^. Figure 1D). These observations raise the question of whether, in the behaving animal, SA to repeated touches is a strong enough effect to be significant above the ‘noise’ of behavioural touch variability or whether it might be a phenomenon only observable when touch variability is unusually low, as under passive whisker stimulation.

### Response attenuation during a touch sequence

**Fig. 2:**
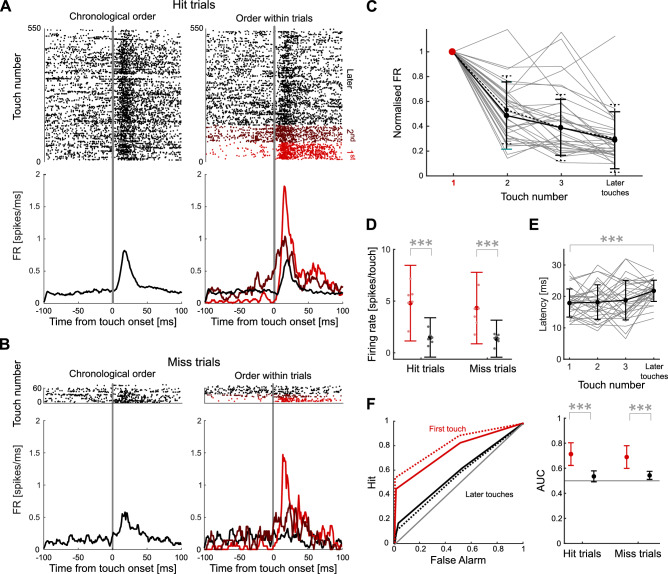
Response attenuation in barrel cortex during touch sequences. (**A**) Raster plots and PSTHs of the neural population recorded during one session (Number of units = 7) divided into hit and miss trials. Left panels show data sorted by chronological order of touches, while right panels show the same data sorted by touch order within trials (first touch, second touch etc.). PSTHs were smoothed (5 ms boxcar) for display only. (**B**) Same as A for miss trials. (**C**) Normalised responses of all units to touch ordered by their occurrence within trials (grey lines). Firing rate was normalised by responses to the first touch. The black solid line represents mean and SD across neurons in hit trials and the dashed black line represents mean and SD across neurons in miss trials. (**D**) Comparison of responses to touches (spike count within 30 ms after onset) during hit and miss trials, across the sample of units. Small dots indicate averages within sessions, large dots show the mean across all units and error bars indicate SD across all units. Asterisks show the results of post-hoc testing (Bonferroni). (**E**) Mean initial latency of the responses of all units to touch ordered by their occurrence within trials (grey lines). Black line shows mean and SD across units. (**F**) Left: Receiver Operating Characteristic (ROC) curve of touch detection for an example unit. Diagonal line represents chance performance (area under ROC curve is 0.5). Solid lines represent ROC for hit trials; dashed lines for miss trials. Right: Area under ROC curve for first and later touches of hit and miss trials across all units. Grey line represents chance performance. For all panels: * indicates *P*-value < 0.05, ** < 0.005, *** < 0.0005.

To determine if SA occurs during active touch, we made extracellular recordings of neural activity (single-unit, N = 33 obtained from 6 sessions from 3 mice) from barrel cortex targeted at column C1 or C2 whilst mice performed the pole detection task. Prior to a bout of touches, firing rate was low (red dots prior to touch onset in Fig. 2A, right). During a touch bout, firing rate remained elevated (black dots prior to touch onset in Fig. 2A, right) and each touch elicited a clear phasic response (Fig. 2A, 2B). For each trial, we classified touches according to whether they were first, second, third, etc. on that trial. We compared responses across touches and between hit and miss trials by 2-way ANOVA (main effects of first vs later touch p < 10^–6^, and hit vs miss trial p = 0.06). We found that the first touch evoked a substantially stronger response than later touches, and that there was a progressive attenuation in response (firing rate) during the course of a series of touches (Fig. 2A-C). We refer to this phenomenon as ‘response attenuation’. Response attenuation was robust across animals: in every mouse, the mean response to second touch was $\le$ 50% of that to first touch (range 42–50%). Responses were similar on error (miss) trials: (ratio of firing rate of first touch on miss vs hit trials 1.06; Fig. 2D), indicating that response attenuation is independent of central processing of trial outcomes. Statistically identical results were obtained when we randomly selected later touches to equalise the number of first-touch and later-touch samples (Number of touches = 123, 2-way ANOVA, main effect of first vs later touch p < 10^–6^; main effect of hit vs miss p = 0.34). The latency of touch responses was greater for later touches (Fig. 2E,* t*-test, mean p-value across units 3 $\cdot$ 10^–4^, Number of units = 33), consistent with previous reports of SA during passive stimulation^10^. We did not observe a significant difference between the width of the peak of the responses to first and later touches (*t*-test across population responses, p = 0.38). The latency of the touch responses was moderately correlated with the pre-touch firing rate (mean Pearson correlation coefficient across units 0.29 ± 0.06, mean p-value = 10^–3^). First touch responses were not only stronger than later touch responses but also more discriminable from spontaneous activity (quantified by area under ROC curve; 2-way ANOVA; main effect of first vs later touches, p < 10^–6^; main effect of hit vs miss, p = 0.53; Fig. 2F). This is in line with the greater pre-touch firing rate for later touches compared to first touch, observed in Fig. 2A (right). Before the first touch in a trial, neurons spike at their baseline level. Pre-touch firing for later touches partly reflects responses to previous touches on a given trial. As detailed below, it is important to distinguish response attenuation from SA, which is just one of several factors that could potentially be responsible.

### Motor adaptation

**Fig. 3:**
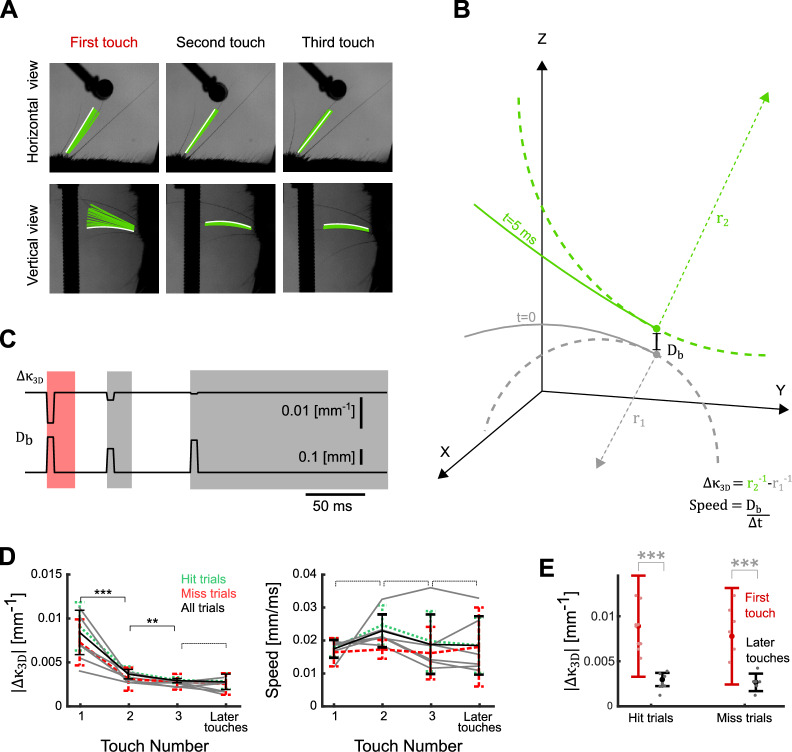
Strength of whisker touch decreases during a touch sequence. (**A**) Single-trial example showing whisker tracking solutions (Bezier curves) projected onto both horizontal and vertical imaging planes. Tracking solution at touch onset is white, whilst solutions for the subsequent 20 ms are green. (**B**) Extraction of whisker shape/displacement parameters from the tracking solutions. Bezier curves describe a whisker at touch onset ($t$ = 0, grey solid curve; circle marks whisker base) and 5 ms later ($t$ = 5, green solid curve). Whisker displacement ($D_{b}$) is the distance between whisker base at $t$ = 0 and that at $t$ = 5, and is proportional to whisker speed. Curvature of the whisker at its base is derived from the length of the radius ($r_{1} ;$ dashed grey line) of the circle (dashed grey curve) whose curvature matches that of the whisker at the base. Dashed green line/curve, with associated radius $r_{2}$, show corresponding data for $t$ = 5. Touch magnitude (${\Delta }\kappa_{3D}$) is the difference in the reciprocals of $r_{1} { }$ and $r_{2}$. $X$, $Y$ and $Z$ axes correspond to the coordinates defined in Fig. 1B). (**C**) ${\Delta }\kappa_{3D}$ and $D_{b}$ (mean of their values in the range $t$ = [0,5] ms) for each of the three touches shown in panel A. Shaded areas indicate touches. (**D**) Left: $\left| {{\Delta }\kappa_{3D} } \right|$ measured as in panel B as a function of touch number for each session (grey lines), averaged across all trials (black), hit trials (green) and miss trials (red); error bars denote SD across trials. Right: Corresponding data for whisker base speed ($D_{b} { }/dt$). E) Comparison of $\left| {{\Delta }\kappa_{3D} } \right|$ for correct and miss trials. Pink dots indicate averages within a session, red dots and error bars are the average and standard deviation over all trials (from all sessions) respectively. Asterisks show results of post-hoc testing (Bonferroni). For all panels: * indicates *P*-value < 0.05, ** < 0.005, *** < 0.0005. Dashed brackets indicate no significant difference.

Although these response attenuation data are consistent with the hypothesis that SA is elicited by active touch during our task, there is a potential alternative explanation. Since the touches in these touch sequences are far from identical (Fig. 1), the decrease in firing rate from first to later touches might potentially be due to physical differences in stimulus strength. To test this possibility, we used 3D whisker tracking to measure both how far, and how fast, the whiskers were displaced during touches, and also how much the whiskers bent during touches (Fig. 3A and 3B). A significant advantage of 3D whisker imaging is that it captures bending and velocity components in the vertical plane that are not visible from the horizontal view alone (see Fig. 3A and 3C, first touch)^28,46^.

We compared whisker speed $D_{b}/\Delta t$ and touch strength (${\Delta }\kappa_{3D}$) (Fig. 3B), computed at the whisker base, of first touches to later touches. We tested the effect of first vs later touch and of hit vs miss trial on these variables by 2-way ANOVA. For first vs later touch, we found that whisker speed was similar but that there was a substantial decrease in ${\Delta }\kappa_{3D}$ (main effect of first vs later touch on ${\Delta }\kappa_{3D}$, p < 10^–6^; on $D_{b}/\Delta t$*,* p = 0.9; Fig. 3D). This indicates that active touch on this task involves pronounced ‘motor adaptation’, whereby animals make progressively weaker pole contact during the course of a trial. The evolution of ${\Delta }\kappa_{3D}$ and $D_{b}/\Delta t$ was similar on hit and miss trials (main effect of hit vs miss on ${\Delta }\kappa_{3D}$, p > 0.8; on $D_{b}/\Delta t$ p = 0.27; Fig. 3D), indicating that, as with the response attenuation reported above, the motor adaptation effect is independent of central processing of trial outcome. Statistically identical results were obtained when we equalised the number of first vs later touch samples (N touches = 123, main effect of first vs later touch on ${\Delta }\kappa_{3D}$, p < 10^–6^; on $D_{b}/\Delta t$, p = 0.62; main effect of hit vs miss on ${\Delta }\kappa_{3D}$, p ≥ 0.3 and on $D_{b}/\Delta t$, p = 0.49). In these analyses, both protraction and retraction touches were included.

Since whisker bending is the best single predictor of the response of whisker mechanoreceptors to touch^38,47–49^, these data imply a decrease in strength of afferent drive to barrel cortex during the course of a trial. This suggests that the attenuation of cortical response reported above (Fig. 2) might not be entirely due to SA but potentially could, either entirely or in part, be an indirect effect of motor adaptation.

### Sensory adaptation

**Fig. 4:**
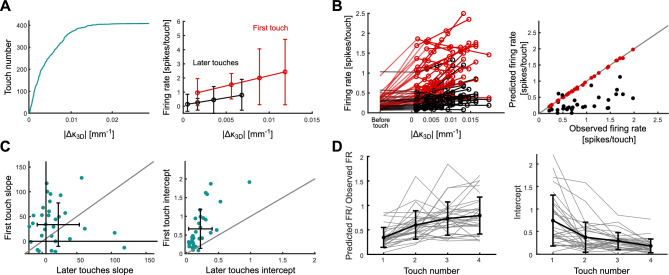
Sensory Adaptation is prominent during active touch. (**A**) Left: sorted values of ${\Delta }\kappa_{3D}$ in an example session. Right: Tuning curves for first and later touches for an example unit recorded during session shown in A**.** (**B**) Left: Tuning curves for first (red) and later (black) touches for all selected units. Responses in the 30 ms interval before touch are shown for comparison. Right: Prediction of response to first touch using tuning curves estimated for later touch (black circles) and first touch respectively (red circles). (**C**) Comparison of tuning curves parameters for first and later touches. Left panel shows slope and right panel shows intercept of tuning curves for all selected units. Error bars indicate mean and standard deviation across units. (**D**) Left: Ratio between firing rate predicted from later touches tuning curve ($N_{touch}$ > 4) and mean observed firing rate for each touch. Grey lines represent mean for each unit and black line represents average across units. Right: Intercept of tuning curves for each touch. Grey lines represent mean for each unit and black line represents average across units.

To determine whether SA does contribute to response attenuation, we developed a method to test for potential changes in SA during the course of a touch sequence. The natural variability in touch reported in Fig. 1, allowed us to estimate tuning curves to touch strength (mean of 71 first touches, 560 later touches per session). Consistent with previous studies^50–53^, we found that barrel cortical units were typically tuned to whisker bending: the greater the bending (${\Delta }\kappa_{3D}$), the greater the firing rate (Fig. 4A). If SA is indeed expressed, the prediction is that tuning curves (firing rate as a function of ${\Delta }\kappa_{3D}$) should shift downwards (units become less sensitive to touch) during the course of a touch sequence. We used linear regression to estimate tuning curves, since we found linearity to be a good approximation within the range of ${\Delta }\kappa_{3D}$ that we were considering (Fig. 4AB).

Figure 4A (right) shows results for an example unit. As predicted by the SA hypothesis, there was a downwards shift in tuning curves from first to later touches. This was a typical result (Fig. 4B). To quantify the extent of this adaptation, we used the tuning curves to estimate and compare the responses of units to first vs later touches, keeping touch strength fixed. Specifically, we compared the actual response of each unit to first touch (red dots in Fig. 4B right) to that predicted by feeding the touch strength (${\Delta }\kappa_{3D}$) of the first touch through the tuning curve estimated for later touches (black dots in Fig. 4B right). For 85% of units, the responses to first touch predicted in this way were significantly lower than the actual responses (*t*-test for each unit ($\alpha$ = 0.05), mean p-value across units = 0.0023)—on average, 59% lower. This was also true on an individual animal basis (mean across mice 57% ± 17). Repeating this analysis for tuning curves estimated for second touch, third touch, etc. in turn showed gradual convergence to an adapted state (Fig. 4D). Both the slope and intercept of the tuning function for later touch decreased significantly compared to those for first touch (Fig. 4C), with the strongest effect observed for the intercept: 93% of units had higher intercept for first touch (mean across mice 97% ± 4); 63% had higher slope for first touch (mean across mice 61% ± 15). On average, the ratio between intercepts from later and first touches was 0.32 (range across individual mice 0.24–0.43). The intercept decreased gradually over the course of later touches (Fig. 4D right). These results indicate that a significant part of the response attenuation from first to later touches observed in Fig. 2 can be explained by a change in neuronal sensitivity. Thus, response attenuation is not simply a consequence of motor adaptation – there is also SA.

To estimate how much of the overall response attenuation in Fig. 2 is due to SA, for each simultaneously recorded population of units, we first determined the overall response attenuation from first to later touch due to the combined effects of motor adaptation and SA (calculated from Fig. 2B). Next, we estimated how much of this decrement could be attributed to the effect of tuning curves alone, keeping touch strength constant (Methods). We found that, on average across the sessions, 73% of the overall attenuation in firing rate is attributable to SA (range across individual mice 51–84%).

In sum, these results indicate that both Sensory Adaptation and Motor Adaptation contribute to the attenuation in firing rate observed during a sequence of whisker-pole touches, with Sensory Adaptation playing the major role.

### Stimulus-specific adaptation

A hallmark of sensory adaptation in studies of anaesthetised animals is that adaptation can be specific to individual whiskers^17,19^. That is, stimulation of a given whisker attenuates responses to subsequent stimulation only of that whisker and not to that of other whiskers (whisker-specific adaptation, WSA). The hypothesis that SA is operative in the actively sensing, behaving animal suggests, therefore, that the changes in tuning curve that we reported above should be whisker-specific. To probe for WSA, we selected all trials in a session in which at least the first two touches were produced by the same whisker – the adapting whisker $W_{ad}$ (C1 or C2) – and at least one later touch (3rd or later) was made by a different whisker-the test whisker $W_{test}$. Simultaneous touches were excluded. WSA predicts that the test stimulus should evoke an unadapted response.

**Fig. 5:**
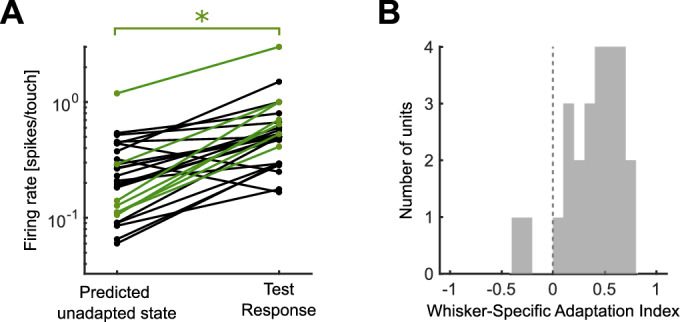
Whisker-specific Adaptation. (**A**) Comparison of response to the test stimulus with that predicted in the adapted state (see main text). Units whose responses were significantly higher than predicted in the adapted state are highlighted in green: * indicates *P*-value < 0.05. (**B**) Histogram of WSA index (I) values across the population.

To test this, we compared the responses ($FR_{test}$) of units to whisker $W_{test}$ touches satisfying the definition of ‘test stimuli’ to responses to whisker $W_{test}$ in the adapted state (that is, responses to the third or later touch of that whisker within a trial, Fig. 5A). To factor out potential differences in touch strength, we measured tuning curves of units to $|{\Delta }\kappa_{3D}$| of that whisker in the adapted state, and used these tuning curves to predict responses to touches of identical $|{\Delta }\kappa_{3D}$| to those measured for the test stimuli ($FR_{predicted}$). WSA predicts that $FR_{test}$ should be greater than $FR_{predicted}$ and that the following WSA index *I* should be positive:

1 $$I = \frac{{FR_{test} - FR_{predicted} }}{{FR_{test} + FR_{predicted} }}$$

Alternatively, if adaptation spreads across whiskers, then *I* should be zero. We found that units had significantly higher $FR_{test}$ compared to $FR_{predicted}$ (Fig. 5B, *t*-test p = 8 × 10^–3^; removing outlier p = 7 × 10^–4^), that 91% of units had positive *I* (Fig. 5B) and that, across the population, *I* was significantly positive (*t*-test; p < 2 × 10^–7^). These results suggest that adaptation in our awake, actively sensing paradigm did exhibit WSA.

## Discussion

Our aim was to investigate the extent to which Sensory Adaptation (SA) is expressed during behaviour, under conditions of active sensation, where the sensory input to the brain is determined by the animal’s own motor actions. Our main finding was that the response of individual neurons in barrel cortex decreased whilst a mouse repeatedly touched an object, and that this was due to two distinct factors: first, a motor adaptation effect, whereby mice tuned their whisking movements so that later touches were mechanically weaker than early ones; second, a sensory adaptation effect, whereby individual neurons became progressively less sensitive to touch. Thus, under conditions where animals are actively sensing the environment, the activity in cortical circuits reflects a delicate interplay between neuronal and behavioural dynamics.

Most previous research on SA used ‘passive sensation’ paradigms, where the animal–usually anaesthetised—is a passive recipient of stimulation applied by the experimenter. The advantage of passive sensation is that the experimenter can deliver a train of identical stimuli and test for SA by comparing responses to them. Although it has long been recognised as important to test for SA in actively sensing, behaving animals, the challenge has been that, during natural active sensation, animals move their sense organs and this makes it problematic to compare responses across repeated stimuli. Our approach was to train mice to perform a task (pole detection) that naturally elicits a stimulus sequence of whisker-pole touches. By tracking the whiskers, we showed that the resulting whisker-object touches are markedly variable in their temporal pattern, duration and strength. To disentangle the effects of this variability on neuronal activity in barrel cortex from that of SA, we used 3D whisker tracking to detect whisker-pole touches and to estimate their strength^28,42,54^. This allowed us to measure how neurons are tuned to touch strength (the primary mechanical variable encoded by neurons in the ascending whisker pathway^38,47–53^ and how this tuning adapts over the course of a touch sequence.

Previous work on SA in the anaesthetised brain or brain slice has found that cortical neurons strongly adapt when stimulated by trains of sensory input with a stimulus rate of a few Hz or more^12,15,30^. However, of the few studies that have been conducted in awake animals, some report that SA is weak^32,34,35^ others that it is substantial^10,20,55^. These differences may reflect cellular/anatomical differences in the recorded neurons, including laminar location and the regions to which the neurons project. The differences may also reflect differences in behaviour such as temporal and mechanical aspects of touch, which is precisely why it is important to study SA under conditions representative of natural behaviour. In particular, since the whisker system is an active sensory system, it is insightful to study SA whilst animals actively control their sensory apparatus to solve behavioural tasks, and to do so in a manner that controls for differences in stimulus strength. Our results indicate that SA is indeed expressed during the course of active whisker exploratory touch sequences. Controlling for differences in mechanical touch strength revealed that neurons were more sensitive to the first touch in a trial than to later touches. Thus, under conditions of active whisker exploration, adaptation significantly affects neuronal responses and tuning.

There are multiple mechanisms which contribute to SA, and SA is known to depend on a host of parameters—stimulus frequency, stimulus magnitude and brain state^2,9,32,56^. This suggests that the mechanisms expressed in any particular experiment are likely to depend on its precise conditions. The first mechanism to be clearly established was synaptic depression in thalamocortical synapses. For SA in the anaesthetised brain, this effect is prominent^15,57^. On the basis that thalamic activity is higher in activated vs quiescent brain states, it was argued that thalamocortical synapses are chronically depressed in the awake brain and, consequently, that there is little scope for thalamocortical synaptic depression to produce SA in the activated brain^32^. However, subsequent research has found that mechanisms involving excitatory-inhibitory balance, neuromodulation by acetylcholine, subcortical adaptation and synchrony-sensitivity also contribute to SA^2,9,15,58–61^. Indeed, a recent study of awake mice, reported that, although SA could not be explained by synaptic depression of thalamocortical synapses, it could be explained by non-linear amplification of thalamic SA, due to the sensitivity of layer 4 cortical neurons to synchrony in thalamic activity^10^. Taken together, these studies suggest that mechanisms different to synaptic depression gain in relative prominence under awake, behaving conditions. Future work should further probe the mechanisms under these conditions. For this, simultaneous recordings of cortical neurons across layers, including of inhibitory interneurons, and their thalamic inputs will help to disentangle the role of thalamic vs cortical mechanisms, potential laminar differences and the role of intracortical inhibition. Additionally, recordings of local field potential might reveal insights into the interplay between sensory adaptation and the level of arousal of the animal. Given that neuromodulators such as acetylcholine can reduce cortical adaptation^61^, and the neuromodulatory input increases with the level of arousal and/or attention^59^, future work should consider its potential impact on SA in behaving animals.

Active sensation is the general principle that animals use motor actions to acquire sensory information and that they select those actions expected to yield the sensory information most useful to their current goal^24,62^. We found that the strength of whisker-pole contact decreased systematically during the course of a trial (motor adaptation). This is reminiscent of the ‘minimal impingement’ strategy whereby whisking amplitude decreases upon contact with an object^63^. It is also consistent with extensive evidence for active sensing in animals with mobile whiskers, including rats, mice and aquatic mammals^26,40,63–67^. Our finding that motor adaptation is similar on correct and incorrect trials suggests that its principal mechanism is insensitive to top-down signals from the brain’s decision-making centres and may involve anatomical loops relatively close to the periphery of the sensorimotor system. Overall, sensory and motor adaptation may be mechanisms to elicit strong neural signals in response to salient, unexpected events.

## Data Availability

Data used to produce the figures in this paper (electrophysiological recordings and tracking of behaviour) is available at Figshare (https://doi.org/10.6084/m9.figshare.22788425.v1). All code used to produce the figures in this paper was developed in MATLAB and is available in our GitHub repository https://github.com/AndreaColinsR/Sensory_Adaptation.
